# Enhancement of the internal quantum efficiency in strongly coupled P3HT-C_60_ organic photovoltaic cells using Fabry–Perot cavities with varied cavity confinement

**DOI:** 10.1515/nanoph-2023-0613

**Published:** 2024-01-08

**Authors:** Lianne M. A. de Jong, Anton Matthijs Berghuis, Mohamed S. Abdelkhalik, Tom P. A. van der Pol, Martijn M. Wienk, Rene A. J. Janssen, Jaime Gómez Rivas

**Affiliations:** Department of Applied Physics and Science Education, Eindhoven Hendrik Casimir Institute, and Institute for Complex Molecular Systems, Eindhoven University of Technology, P.O. Box 513, 5600 MB Eindhoven, The Netherlands; Department of Chemical Engineering and Chemistry, and Institute for Complex Molecular Systems, Eindhoven University of Technology, P.O. Box 513, 5600 MB Eindhoven, The Netherlands

**Keywords:** strong coupling, organic photovoltaics, Fabry Perot cavities

## Abstract

The short exciton diffusion length in organic semiconductors results in a strong dependence of the conversion efficiency of organic photovoltaic (OPV) cells on the morphology of the donor-acceptor bulk-heterojunction blend. Strong light–matter coupling provides a way to circumvent this dependence by combining the favorable properties of light and matter via the formation of hybrid exciton–polaritons. By strongly coupling excitons in P3HT-C_60_ OPV cells to Fabry–Perot optical cavity modes, exciton-polaritons are formed with increased propagation lengths. We exploit these exciton–polaritons to enhance the internal quantum efficiency of the cells, determined from the external quantum efficiency and the absorptance. Additionally, we find a consistent decrease in the Urbach energy for the strongly coupled cells, which indicates the reduction of energetic disorder due to the delocalization of exciton–polaritons in the optical cavity.

## 1 Introduction

Organic photovoltaic (OPV) is an emerging technology with unique benefits and great potential for future applications due to the low cost, flexibility, and transparency of organic solar cells [1], [2]. Recent developments within the field of OPV have led to the achievement of power conversion efficiencies of over 19 % [3]. OPV cells are based on electron-donating and electron-accepting organic semiconductors. An important parameter determining the efficiency of these OPV cells is the diffusion of excitons to the donor–acceptor interface, where they can dissociate into holes and electrons to generate a current. Frenkel excitons in organic semiconductors have large binding energies and are localized onto only a few molecular sites. As a result, these excitons move via a diffusive process of incoherent hopping [4]. This hopping process, in combination with short exciton lifetimes, greatly limits exciton transport in OPVs and results in diffusion lengths of only a few nanometers. To partly overcome the problem of short diffusion lengths, bulk hetero-junctions (BHJ) are typically employed instead of bi-layer junctions [4]. The average distance that excitons have to travel in BHJ solar cells before they reach the interface is decreased by forming donor-acceptor blends. However, this configuration also has disadvantages in the form of geometrical constraints, such as an increased surface recombination and the formation of islands, which reduce charge percolation pathways [5].

Alternatively, instead of reducing the average distance to donor–acceptor interfaces, one could aim to increase the exciton diffusion length. Previous research has shown that excitons in organic materials, when placed in an optical cavity, can strongly couple with the confined optical modes, leading to the formation of new hybrid quasi-particles, called exciton–polaritons (EPs) [6], [7]. EPs combine the properties of excitons with the delocalized nature of the optical mode. Due to this delocalized nature, significantly increased propagation lengths of EPs compared to excitons and enhanced charge transfer have been both theoretically predicted [8]–[10] and experimentally demonstrated [11]–[21].

Herein, we demonstrate an enhancement of the internal quantum efficiency (IQE) of OPV solar cells by EPs. This demonstration is achieved by strongly coupling excitons in poly(3-hexylthiophene) (P3HT) to a Fabry–Perot cavity optical mode in a bilayer junction cell. It is expected that bilayer junction cells will benefit most from an increased exciton diffusion length by strong coupling, as BHJ cells are designed such that their charge separation efficiency is already close to unity. Nevertheless, also BHJ cells may potentially benefit from strong light–matter coupling, as this will allow for a re-optimization of the BHJ mixing structure.

Recent works have discussed the application of strong light–matter coupling in organic photodiodes and solar cells using Fabry–Perot (FP) cavities [22]–[24]. Eizner and co-workers have demonstrated an extended responsivity of organic photodiodes in the infrared by the energy shift associated with the formation of polaritons in the ultra-strong light–matter coupling regime [22]. By varying the angle of incidence and the device thickness, they have shown a very rich response, with external and internal quantum efficiencies at the polariton wavelengths that can be enhanced or reduced compared to a reference. A red-shift of the absorption edge in solar absorbers has also been reported by Bujalance and co-workers by ultra-strong light–matter coupling [25]. This work was limited to optical experiments and no electrical characterization was presented. Photodetectors have been also realized by Mischok and co-workers by strong coupling to carbon nanotubes in the near-IR [26]. Wang and co-workers have demonstrated an impressive up to 50-fold enhancement of the IQE of photodiodes under strong light–matter coupling [23], and they have attributed this enhancement to the improved exciton-polariton transport. However, the absolute performance of these photodiodes is very low, with an external quantum efficiency (EQE) of only 0.01 % and no measurable open-circuit voltage. These works raise the question if an improved performance can also be reached in more competitive OPVs, with the ultimate goal of improving state-of-the-art OPV efficiencies. Nikolis and co-workers have demonstrated the reduction of voltage losses in strongly coupled OPV cells [24]. This loss reduction is related to the steepness of the absorption edge, which is represented by the Urbach energy and accounts for the disorder in the energy levels of the excited states. We demonstrate here a reduction of the Urbach energy in strongly coupled OPV cells, which we investigate for different cavity lengths and coupling strengths, and hypothesize that this reduction is the result of motional narrowing in delocalized EPs. Furthermore, we showcase in this manuscript an enhancement of the IQE for strongly coupled OPV cells with an EQE performance up to 10 %, which we relate to increased EP harvesting through their delocalized nature and increased propagation length.

### 1.1 Sample design

**Figure 1:**
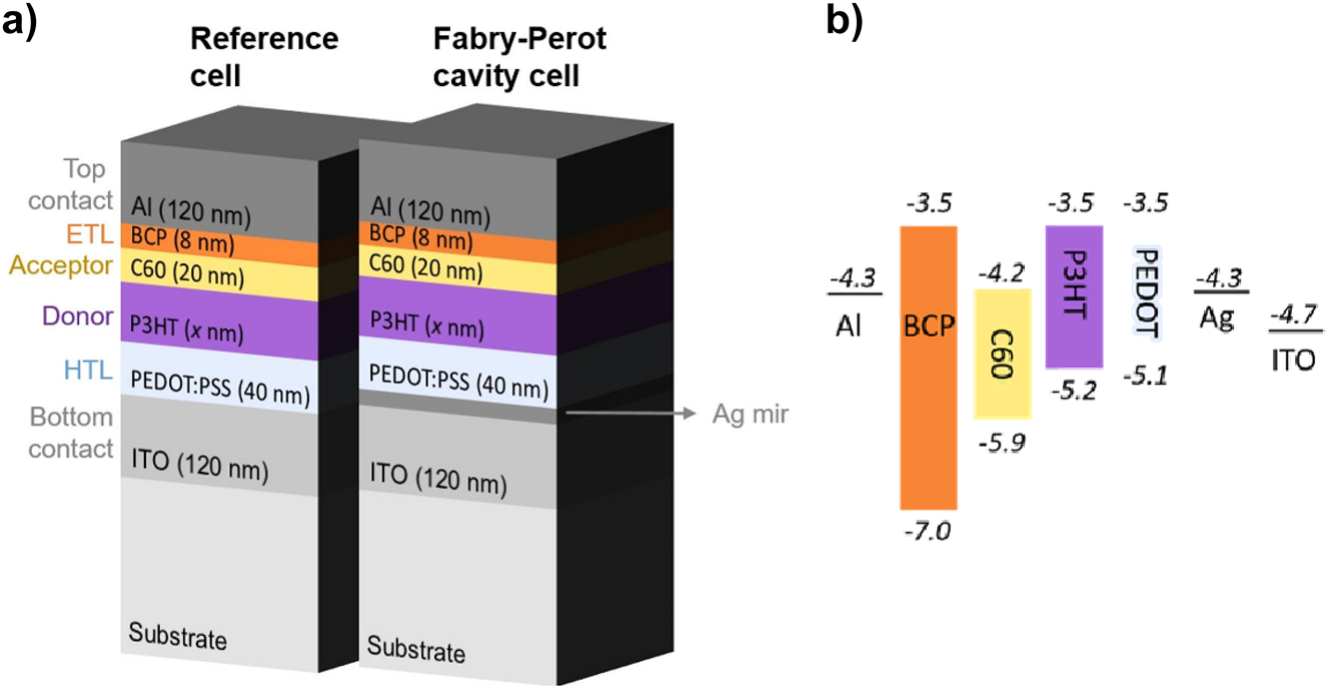
Devices’ structure and energy level diagram. (a) Device structure of the reference P3HT-C_60_ cells and of the FP cavity cells exhibiting strong light–matter coupling. To vary the cavity length, 60 and 80 nm P3HT layer thicknesses have been investigated. The thickness of the Ag film is varied between 10 and 30 nm to change the losses of the FP cavity mode. (b) Energy level diagram of each layer of the OPV cell. The values are retrieved from Refs. [27], [28].

To demonstrate the potential of strong light–matter coupling in OPVs, we have investigated a bilayer configuration with P3HT as donor and buckminsterfullerene (C_60_) as acceptor. The investigated OPV cells are illustrated in Figure 1(a). We use a multilayer glass/ITO (120 nm)/PEDOT:PSS (40 nm)/P3HT/C_60_ (20 nm)/BCP (8 nm)/Al (100 nm) device architecture as a reference, while the Fabry–Perot OPV cells were fabricated with an additional Ag film on top of the ITO layer (for a detailed description see Section 4). We used a highly transmissive indium tin oxide (ITO) bottom contact and a reflective aluminum (Al) top contact. The PEDOT:PSS is used as a hole-transport layer (HTL) and bathocuproine (BCP) is used as electron transport layer (ETL). The thickness of the P3HT layer is varied between 60 and 80 nm. The thickness of the Ag film that is added to define the Fabry–Perot cavity and achieve strong coupling is varied between 10 and 30 nm. This Ag film thickness determines the quality of the cavity and affects the transmission of the light into the OPV cell. We chose to focus on thin Ag films to maximize the amount of light entering the cell, as more light leads to an increase in optically excited electrons, which are needed to generate current and create a working OPV cell. The energy diagram of this OPV stack is shown in Figure 1(b).

## 2 Results and discussion

### 2.1 Dispersion measurements

**Figure 2:**
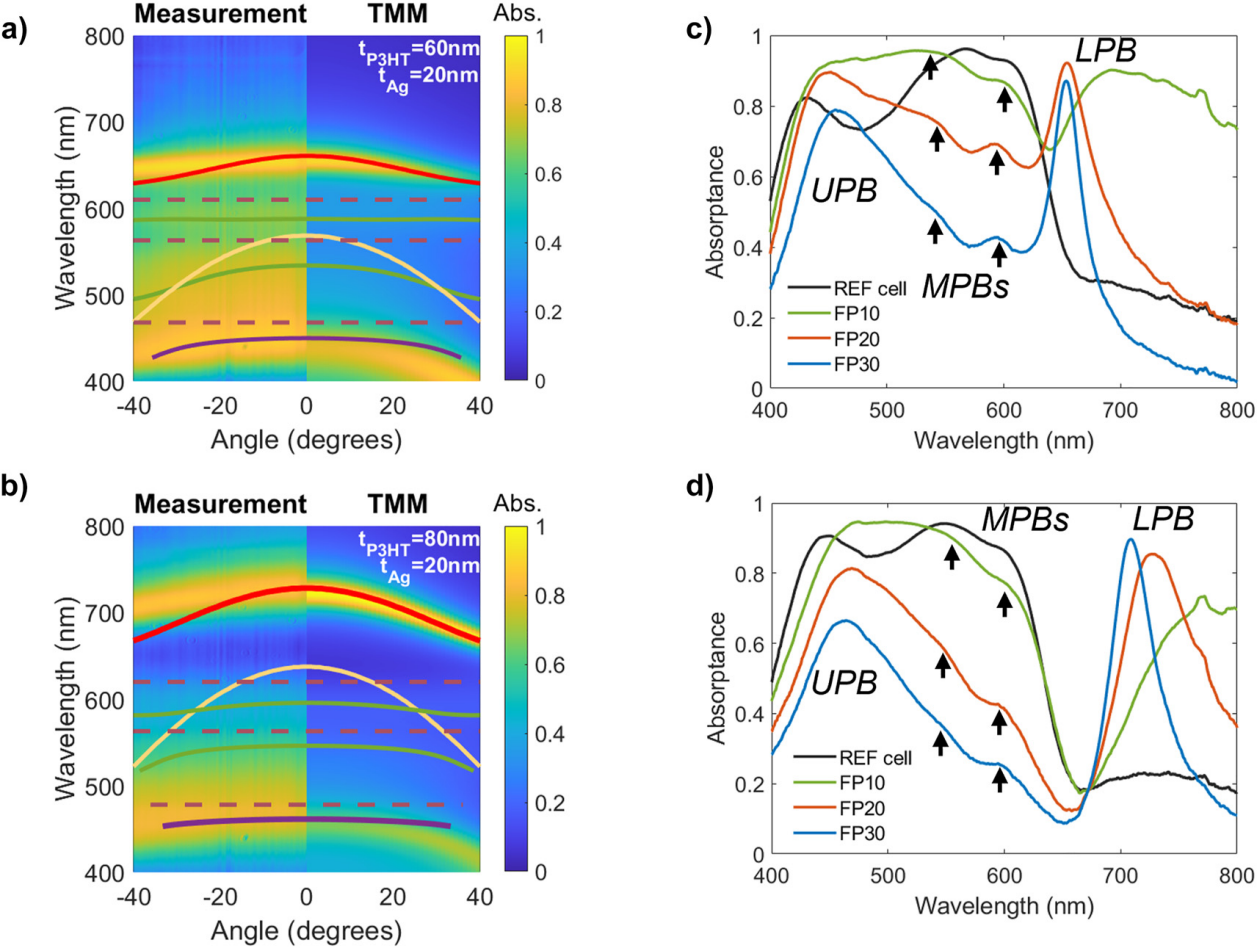
Angular absorptance dispersion of the FP cavity cells with a P3HT thickness of 60 nm (a) and 80 nm (b), and an Ag film of 20 nm thickness measured in a Fourier microscope (left panels) and calculated using TMM (right panels). The UPB, MPBs, and LPB are represented by purple, green, and red curves, and the uncoupled FP mode and exciton levels are shown by the yellow curves and dotted lines, respectively. (c) and (d) are the measured absorptance at normal incidence for OPV cells with a P3HT layer of 60 nm and 80 nm, respectively. These panels show the absorptance of the reference cells and of the FP cells with Ag films of thickness 10 (FP10), 20 (FP20), and 30 nm (FP30).

To characterize the coupling strength of excitons in P3HT with the FP cavity modes, the absorptance of the P3HT-C_60_ OPV cells has been measured and calculated as a function of wavelength and angle of incidence. The measurements were done using a Fourier spectroscopy setup in reflection mode (see Methods), since the cells do not transmit light, the absorptance is defined as 1 − *R*, where *R* is the reflectance. The calculations were performed with the transfer matrix method (TMM, see Section 4). The results of the dispersion of the absorptance (i.e., absorptance spectra as a function of the angle of incidence) of strongly coupled OPV cells are presented in Figure 2(a) and (b) for a P3HT layer thickness of 60 and 80 nm, respectively, and an Ag film thickness of 20 nm (see Supporting Information (SI), Section S1 for the dispersion of the other Ag thicknesses). The dispersion is fitted using a four coupled oscillator model for the bare states [29] (see Section 4). The bare Fabry–Perot resonances, indicated by yellow curves in Figure 2(a) and (b), are determined with TMM calculations of the FP cavity structure without excitons, i.e., without absorption in the P3HT and C_60_ layers. The uncoupled exciton energies are retrieved from the P3HT absorption spectrum without cavity effects, showing three distinct exciton peaks (see SI Section S2). Unlike the P3HT excitons, the C60 excitons were found not to couple to the optical mode. The main reason for the lack of coupling of the C60 excitons is the location of the C60 layer, which is further away from the cavity field maximum (see SI Section S5). As a result, the number of generated excitons in C60 is small, resulting in a weak interaction between C60 excitons and the bare cavity mode. Because three exciton transitions (indicated by the horizontal dashed lines in Figure 2(a)) couple to one optical mode, four polariton bands are formed: the lower polariton band (LPB, indicated by the red curves in Figure 2(a) and (b)), two middle polariton bands (MPBs, indicated by the green curves), and the upper polariton band (UPB, indicated by the purple curve). Only the LPB and UPB are clearly visible in the dispersion measurements and simulations, while the MPBs are less pronounced due to the larger absorption of the P3HT at these wavelengths. These polariton bands red-shift with increasing cavity thickness, as expected by the redshift of the cavity mode.

The absorptance spectra measured at normal incidence are given in Figure 2(c) and (d) for the cells with a 60 and 80 nm thick P3HT layer, respectively, and for different thicknesses of the Ag layer (10, 20, and 30 nm). In these figures, the difference between the absorptance in the reference cell and the strongly coupled cells can be compared. We see a pronounced reshaping of the absorptance spectrum, with the formation of multiple peaks, located at the polariton wavelengths. The LPB and UPB are clearly visible for the cells with the 20 and 30 nm thick Ag layers. Also the MPBs can be appreciated as small bumps in the absorptance spectra, as indicated with the black arrows in Figure 2(c) and (d). The Rabi-splitting, defined by the smallest separation in energy between the measured LPB and UPB peaks, is 0.81 ± 0.07 eV for a P3HT layer of 60 nm and 0.92 ± 0.05 eV for a P3HT layer of 80 nm in the case of an Ag mirror thickness of 30 nm, which corresponds to the highest cavity quality investigated. The measured Rabi-splitting is larger than the full width at half maximum of the bare FP resonance, approximated to be 0.08 eV based on TMM calculations, and of the uncoupled excitons, with varying FWHM between 0.5 eV and 0.1 eV (see SI Section S2). Because of this larger Rabi-splitting, it can be concluded these organic solar cells are in the strong coupling regime. Additionally, we see in Figure 2(c) and (d) that the polariton peaks become increasingly narrow for thicker Ag films, which is the result of the lower radiation losses from the Fabry–Perot cavity with increasing cavity quality.

### 2.2 Current density-voltage measurements

**Figure 3:**
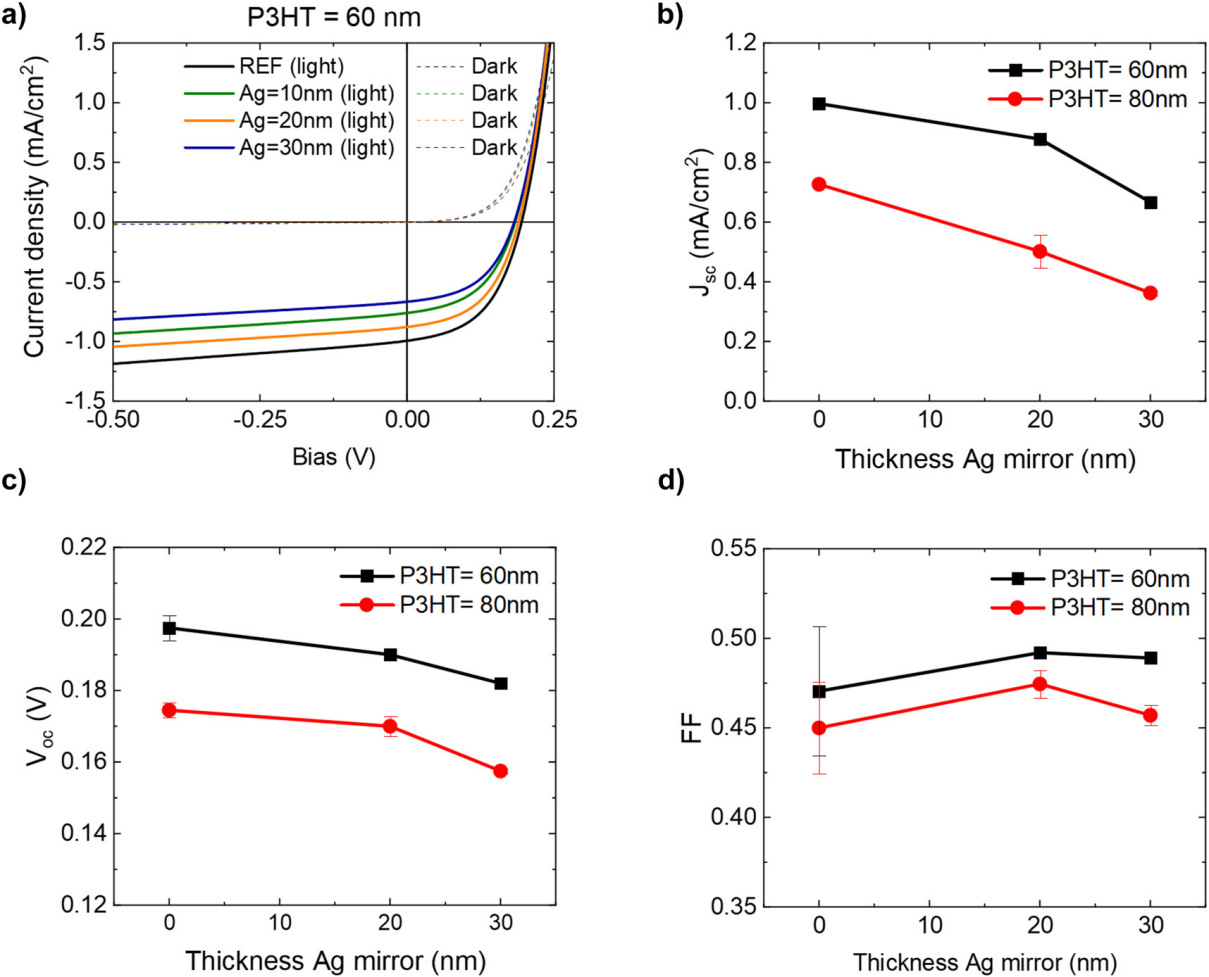
Optoelectronic characterization. (a) Measured *JV*-curves for cells with a P3HT layer thickness of 60 nm and different Ag mirror thickness, showing both the light *JV*-curve (measured under standard solar conditions) and the dark *JV*-curve. (b) Retrieved short-circuit current density, (c) open-circuit voltage, and (d) fill factor plotted as a function of the silver film thickness for cells with a P3HT thickness of 60 and 80 nm.

In order to analyze the change in efficiency of the strongly coupled P3HT-C_60_ solar cells, their opto-electrical properties have been measured, i.e., the current density–voltage (*JV*) curves and EQE spectra. The measured light and dark *JV*-curves show a clear diode behavior, as shown in Figure 3a for the cells with a P3HT layer thickness of 60 nm (see SI Section S3 for the *JV*-curves of 80 nm P3HT solar cell). From the *JV*-curves, the short-circuit current density (*J*_sc_), open-circuit voltage (*V*_oc_), and fill factor (*FF*) can be determined. These three parameters define the maximum power that the cell can generate. The efficiency of the cell is given by

(1) $$\eta =\frac{J_{\text{sc}}V_{\text{oc}}FF}{P_{\text{in}}},$$

with *P*_in_ the incident irradiance. The *J*_sc_, *V*_oc_, and *FF* are plotted as a function of the Ag mirror thickness for the *t*_P3HT_ = 60 and 80 nm cells in Figure 3(b)–(d), respectively. Here, a zero Ag film thickness refers to the reference cells. In total four substrates have been measured with four contacted cells on each substrate that all have a different Ag film thickness. Two substrates have a P3HT thickness of 60 nm and two substrates have a P3HT thickness of 80 nm. The uncertainty is determined by the standard deviation between the measurement results of the different substrates for the same cavity designs. In case a cell on one of the substrates was broken, no uncertainty can be given. The measurements for the cells with a 10 nm Ag mirror have been omitted in Figure 3. The reason for this omission is the poor Ag film quality due to the formation of islands when thermally evaporating thin metal films (see SI Section S4). Therefore, these cells are not considered in further analysis.

Figure 3(b) shows that the *J*_sc_ reduces with Ag film thickness, which is a direct result of the reduced absorption in the active layers due to the limited transmission through the Ag films. The *V*_oc_, plotted in Figure 3(c), also shows a small reduction with increasing film thickness. This reduction is the result of the lower *J*_sc_ as can be estimated from

(2) $$\Delta V_{\text{oc}}=n_{\text{id}}\frac{kT}{q}\ln\left(\frac{J_{\text{sc}}}{J_{\text{sc0}}}\right),$$

where *J*_sc0_ is the short-circuit current density when no Ag film is present, *n*_id_ is the ideality factor of the diode, which can be assumed close to unity when the main loses are radiative or occur via trapped states outside of the junction, *k*_b_ is Boltzmann’s constant, *T* is the absolute temperature, and *q* is the fundamental charge. The loss in *V*_oc_ of cavity cells with 30 nm Ag mirrors of 16 meV and 17 meV for the 60 and 80 nm P3HT layers respectively, are close to the calculated reduction of the *V*_oc_ from the reduced *J*_sc_, being 11 meV and 18 meV. Despite the reduction of *V*_oc_ and *J*_sc_ with the thickness of the Ag film, the *FF* remains remarkably constant, as can be appreciated in Figure 3(d). The different cell parameters are listed in Table 1.

**Table 1:** Short-circuit current density (*J*_sc_), open-circuit voltage *V*_oc_, fill factor *FF* and maximum power generated by the cell, given by the product of *J*_sc_, *V*_oc_, and *FF*.

Ag thickness (nm)	*J*_sc_ (mA/cm^2^)	*V*_oc_ (V)	*FF*	Maximum power (mW/cm^2^)
**P3HT (60 nm)**				
0	1.00	0.198	0.47	0.09
20	0.878	0.190	0.492	0.08
30	0.667	0.182	0.489	0.06
**P3HT (80 nm)**				
0	0.73	0.175	0.45	0.06
20	0.50	0.170	0.47	0.04
30	0.36	0.158	0.46	0.03

### 2.3 External and internal quantum efficiency

**Figure 4:**
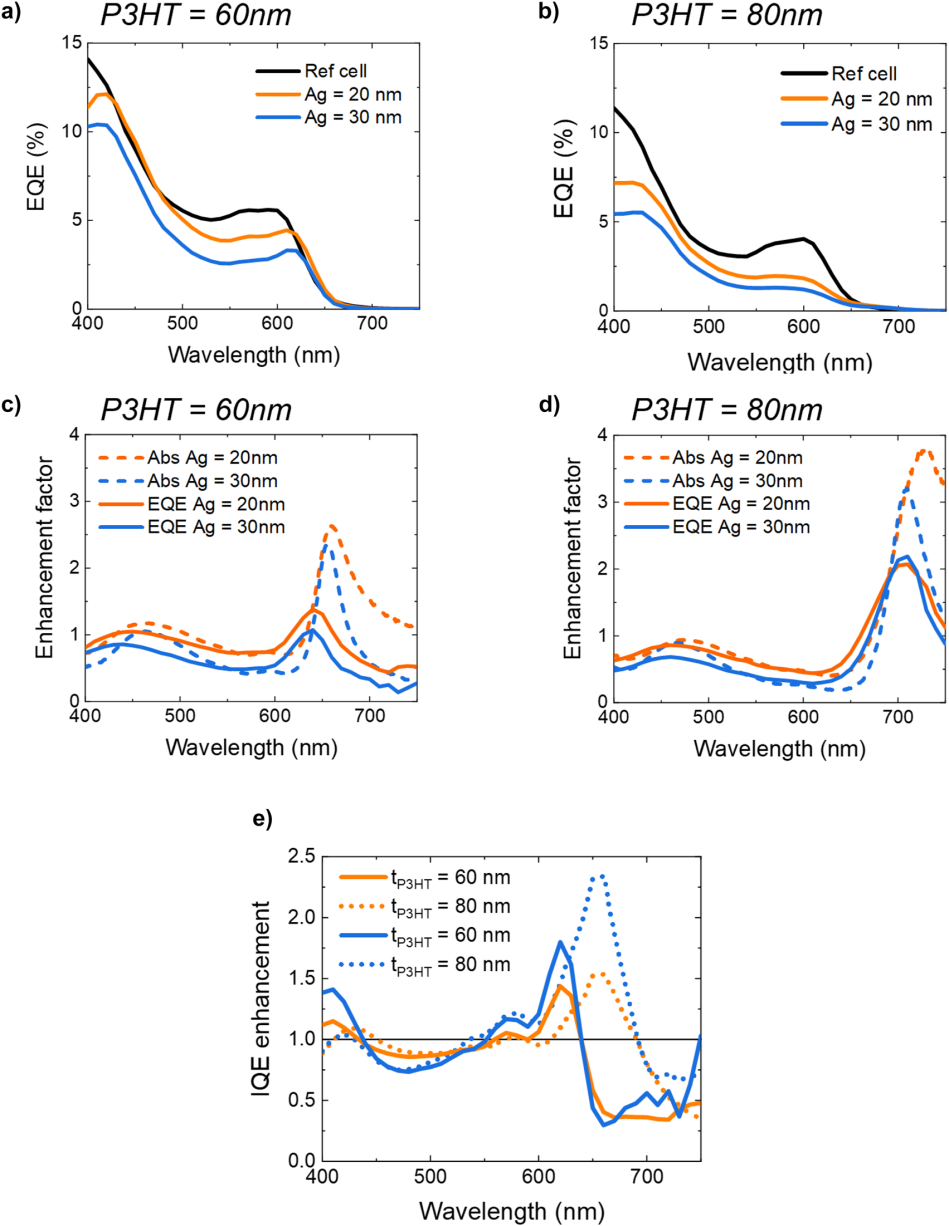
Measured EQE spectra of the reference and FP cavity cells with varying Ag film thickness and a P3HT thickness of 60 nm (a) and 80 nm (b). EQE_en_ and Abs_en_ of FP cavity cells with a 30 nm Ag film for a P3HT thickness of 60 nm (c) and 80 nm (d). The peaks of the EQE_en_ and Abs_en_ are located around the polariton band wavelengths. (e) IQE_en_ of FP cavity cells for P3HT thicknesses of 60 nm (solid curves) and 80 nm (dotted curves) and Ag films 20 nm (orange) and 30 nm (blue).

The external quantum efficiency spectra of the P3HT-C_60_ reference and FP cells are shown in Figure 4(a) and (b) for the P3HT layer thickness of 60 and 80 nm, respectively. The EQE is determined by the ratio of electrons extracted from the OPV cell to the number of incident photons. To visualize the reshaping of the EQE spectra of the strongly coupled cells, we have calculated the EQE enhancement (EQE_en_) by dividing the EQE spectra of the strongly coupled cells through the EQE spectra of the reference cells. The results are plotted in Figure 4(c) and (d) for the 20 and 30 nm Ag mirror and P3HT layer thicknesses of 60 and 80 nm, respectively, together with the absorptance enhancement (Abs_en_) spectra. Abs_en_ is calculated by dividing the absorptance of the strongly coupled cells through the absorptance of the reference cells at normal incidence. Both the EQE_en_ and Abs_en_ spectra show clear peaks close to the LPB and UPB wavelengths.

The measured EQE_en_ and Abs_en_ of the strongly coupled P3HT-C_60_ cells are used to determine the internal quantum efficiency enhancement (IQE_en_), with is defined as

(3) $${\text{IQE}}_{\text{en}}=\frac{{\text{EQE}}_{\text{en}}}{{\text{Abs}}_{\text{en}}}.$$

The IQE_en_ spectra are plotted in Figure 4(e) for the two different P3HT thicknesses and Ag film thicknesses. The figure shows a significant wavelength dependent enhancement of the internal quantum efficiency in the case of the strongly coupled cells. We observe that, similar to the EQE enhancement, also the IQE enhancement is highest around the energy of the polaritons, which indicates that resonant pumping of the polaritons leads to a more efficient propagation to a donor-acceptor interface due to the longer propagation length of resonantly pumped polaritons [30]. Note that although the largest EQE enhancement occurred at the polariton energies, the even larger absorption enhancement in the mirrors at slightly red-shifted energies (see SI, Section S5) results in a blueshift of the IQE enhancement peaks compared to the polariton energies. The peaks corresponding to the LPB and UPB are most clearly visible, but also small bumps in the IQE spectra related to the MPBs can be distinguished. This enhancement increases both with P3HT thickness, which in these cells also corresponds to a larger Rabi-splitting, and with the Ag film thickness, which is directly related to the quality of the cavity. The IQE_en_ spectra also show dips, where the internal efficiency of the strongly coupled cells is lower compared to the reference cells. These dips are slighty red shifted compared to the polariton wavelengths and are the result of an underestimation of IQE_en_ due to the fact that it is calculated using the total absorptance from the cell, which includes absorption in layers that do not contribute to the EQE, e.g., absorption in the Ag layer or the Al contact. Even though the absorption coefficient in these layers is much lower than the absorption in the active layers, the absorption is not negligible due to the large field enhancement at the polariton energies (see SI, Section S5). It should be noted that correcting for absorption in the Ag layer and the Al contact by measuring absorptance in a cell without these inactive layers is unfeasible. This impossibility arises from the altered field distribution and, consequently, varying absorption within a cell lacking these layers.

### 2.4 Reduction of disorder

Finally, we have evaluated the steepness of the absorption edge of the strongly coupled P3HT-C_60_ cells. Ideally, the absorption edge resembles a step function with no absorption of photons with energies below the optical bandgap of the semiconductor. The steepness of the absorption edge is related to the disorder in the semiconductor [31]–[33], and is characterized by the Urbach energy *U*_e_, which can be determined by fitting the slope of the low energy EQE tail with an exponential decay function [31]–[33],

(4) $${\text{EQE}}_{\text{tail}}\left(E\right)\propto \exp\left(-\frac{E}{E_{\text{u}}}\right).$$

**Figure 5:**
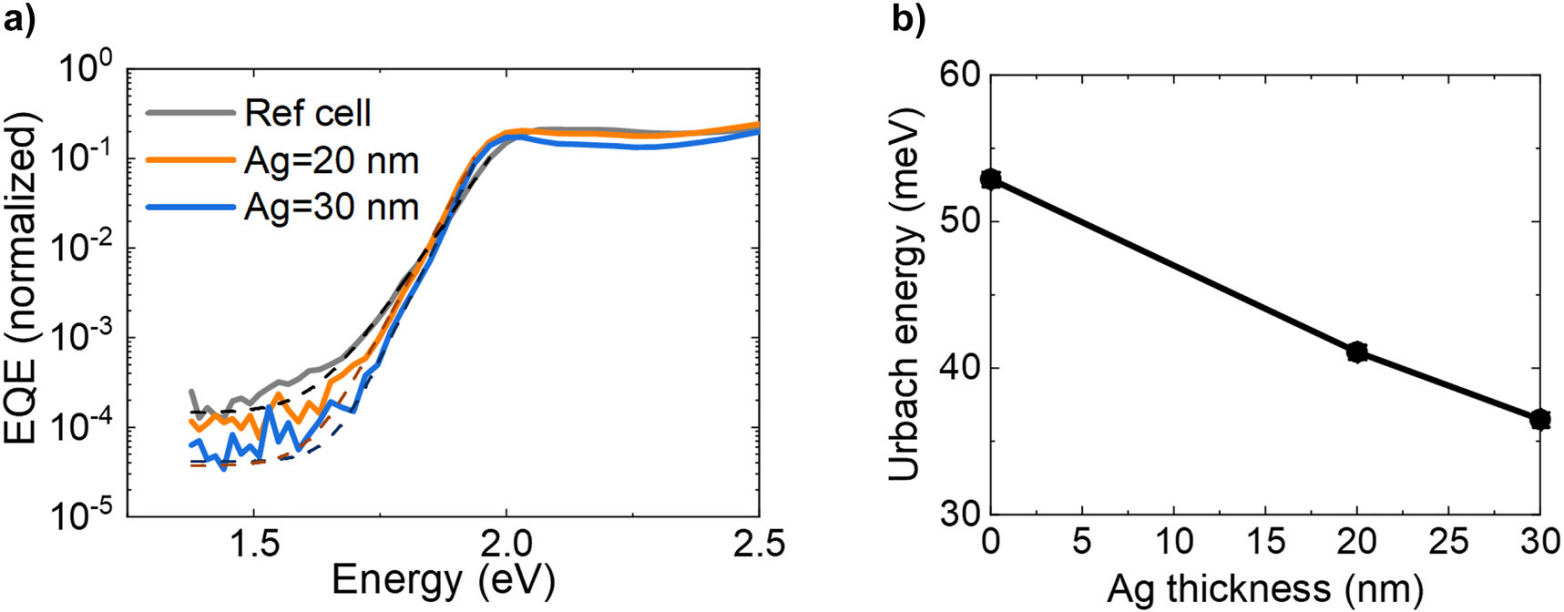
Steepening of the absorption edge for strongly coupled FP cells. (a) Low energy tail of the measured EQE spectra cells of 60 nm P3HT and with different Ag mirror thicknesses. The dashed curves show the exponential decay fits. (b) Urbach energy retrieved from the exponential decay fit and plotted as a function of Ag film thickness.

In Ref. [24], it has been shown that by using strong light–matter coupling in OPV SubNC/Cl_6_-PhOSubPc devices, Urbach energies comparable to those of crystalline inorganic semiconductors could be reached, which corresponded to a reduction in Urbach energy of about 30 % compared to the reference OPV cells. We have measured similar reductions in the Urbach energy for our P3HT-C_60_ cells. The fitted low energy EQE tails, normalized around 2 eV, of the P3HT-C_60_ cells are shown in Figure 5(a) for a P3HT layer of 60 nm. The EQE spectra of the OPV cells with 80 nm P3HT thicknesses cannot be fitted with the exponential decay function, as the LPB energy for these cells is within the low energy tail, reshaping it and making the evaluation of the slope difficult (see SI Section S6).

The fitted Urbach energies are plotted as a function of Ag film thickness in Figure 5b and show a clear decreasing trend with this thickness. This reduction of the Urbach energy shows that it is correlated to the extent to which the light is confined in the cavity. For these *t*_P3HT_ = 60 nm OPV cells, we find an Urbach energy reduction of 31 % in the case of *t*_Ag_ = 30 nm. We have measured similar trends of Urbach energy reduction for strongly coupled cells with a P3HT thickness of 70 nm (see SI Section S6). We hypothesize that this significant reduction in Urbach energy in the case of strong light–matter coupling is the result of motional narrowing of the exciton energy distribution at the energy of the lower polariton band and the spatial averaging of the disorder due to the improved diffusion and the delocalized nature of exciton-polaritons [34]–[36].

## 3 Conclusions

We have demonstrated strong light–matter coupling in OPV cells based on a P3HT-C_60_ bilayer junction using a Fabry–Perot cavity that was fabricated by adding a thin Ag film on top of the ITO contact. The strongly coupled OPV cells show an improvement of the IQE compared to the P3HT-C_60_ reference cells. We have also shown that the IQE enhancement depends both on the cavity thickness and quality, which were varied by changing the thickness of the P3HT layer and Ag film. The largest IQE enhancement was found for the cells with the highest FP-cavity quality and largest P3HT thickness, which also showed the largest Rabi-splitting. Finally, we have evaluated the Urbach tails and found a clear reduction in Urbach energy by strong light–matter coupling. This reduction in Urbach energy and the improved IQE of cells operating in the strong coupling regime indicate the delocalized nature of exciton–polaritons and enhanced exciton–polariton diffusion due to their hybrid character and photonic content. These results illustrate the potential impact of strong light–matter coupling to improve the performance of real optoelectronic devices.

## 4 Methods

### 4.1 Sample fabrication

Patterned ITO substrates were subsequently cleaned in acetone by ultrasonication, then rubbed with sodium dodecylsulfate solution in demi-water, rinsed with demi-water, cleaned with isopropanol, and cleaned for 30 min in UV-ozone cleaner. For the cavity cells, a silver mirror was deposited using a metal evaporator. On top of that, a PEDOT:PSS layer was spin coated in ambient conditions. Then the sample was inserted in a nitrogen filled glovebox where the P3HT was spin coated from a solution of 1,2-dichlorobenzene. Then the C_60_ and BCP films and the aluminum back reflector were deposited in an evaporator.

### 4.2 Absorption measurements

The absorption measurements are done in a Fourier microscope. The sample is illuminated using a white lamp (Fiber-coupled xenon light source from Thorlabs) through a 60 × 0.7 NA objective lens (Nikon CFI S plan Fluor). The reflected light by the sample is collected using the same objective and focused by a lens with a focal length *f* = 200 mm into a real plane image. A pinhole at this real plane is used to filter out unwanted reflections. A second lens with *f* = 150 mm is used to image the back focal plane of the objective at the entrance of a spectrometer (Princeton Instruments SP2300) with an imaging camera (Princeton Instruments ProEM:512).

### 4.3 Current density – voltage measurements

The *JV*-curves are measured using a solar simulator and a four probe system. Light from a tungsten-halogen lamp goes through two filters (Schott GG385 UV filter and a Hoya LB120 daylight filter) to simulate AM1.5G conditions. For the electrical contact, two probes are placed on the positive and negative contact of the OPV cell. A Keithly 2400 SourceMeter is used to apply voltage bias and measure the current.

### 4.4 External quantum efficiency spectra

The EQE spectra are measured using a home-built setup for illuminating the sample and measuring the generated current. A tungsten-halogen lamp (50 W) is used as light source. The light is mechanically chopped (Stanford Research systems SR540) and dispersed by a monochromator (Oriel Cornerstone 130) through an aperture of 0.0314 cm^2^ in order to have a fixed illumination area for all cells. The cells are kept in a nitrogen filled sealed box. The current is measured using a low-noise current pre-amplifier (Stanford Research Systems SR570) and a lock-in amplifier (Stanford Research Systems SR830). The setup is calibrated using a Silicon reference cell.

### 4.5 Transfer matrix method (TMM) calculations

The TMM model is a powerful and fast method to calculate transmission, reflection and absorption of a multilayer system based on Maxwell equations. The multilayer system is represented by a discrete amount of interfaces separated by bulk material. The layers are assumed to have a finite thickness, but stretch out infinitely in the 2D plane. Each interface in the multilayer stack is described by a 2 × 2 matrix, including the reflection and transmission of waves going from left to right and from right to left. Absorption in the layers is represented by an additional 2 × 2 matrix, with diagonal terms describing a plane wave traveling through the absorbing medium. Multiplying these matrix elements gives the total transmission, reflection and absorption. The material data for the P3HT and C_60_ layers used in the TMM simulations are obtained from ellipsometry measurements (see SI, Section S7).

### 4.6 Coupled oscillator model

A coupled oscillator model is used to fit the polariton bands and obtain the Rabi splitting. The Hamiltonian used to describe the coupled damped system is:

(5) $$H=\left[\begin{array}{cccc} E_{\text{FP}}-i\gamma_{\text{FP}} & g_{\text{e}1} & g_{\text{e}2} & g_{\text{e}3}\\ g_{\text{e}1} & E_{\text{e}1}-i\gamma_{\text{e}1} & 0 & 0\\ g_{\text{e}2} & 0 & E_{\text{e}2}-i\gamma_{\text{e}2} & 0\\ g_{\text{e}3} & 0 & 0 & E_{\text{e}3}-i\gamma_{\text{e}3} \end{array}\right].$$

In this model, we consider the coupling of the three excitons in P3HT (see SI Section S2) to the cavity mode and neglect the coupling between excitonic transitions. The resonance energy and cavity losses are determined beforehand by fitting the resonance peaks with a Gaussian function. The energies of the excitons are 2.03 eV, 2.20 eV and 2.65 eV with losses of 100 meV, 400 meV, and 400 meV for *E*_e1_, *E*_e2_, and *E*_e3_ respectively. The coupling strength between cavity and exciton(s) are used as fitting parameters and found to be 140 meV, 240 meV, and 260 meV for *g*_e1_, *g*_e2_, and *g*_e3_, respectively. The polariton band frequencies are found by solving the determinant of the Hamiltonian det(***H*** − *λ* ***I***).

## Supporting Information available

– S1. Additional dispersion measurements of the absorptance of the OPV cells.
– S2. Bare Fabry–Perot resonances and uncoupled excitons.
– S3. *JV*-curves.
– S4. Scanning electron microscope image of thin film Ag (10 nm).
– S5. Electric field distributions.
– S6. Low energy absorption tails.
– S7. Optical constants of P3HT.

## Supplementary Material

Supplementary Material Details
