# Supplementary material for: Enhancement of the internal quantum efficiency in strongly coupled P3HT-C60 organic photovoltaic cells using Fabry–Perot cavities with varied cavity confinement
Source: Nanophotonics. 2024 Jan 8;13(14):2531–40. doi: 10.1515/nanoph-2023-0613 (PMC11147493; doi:10.1515/nanoph-2023-0613)
Supplement: Supplementary file 1 — Supplementary Material Details [file j_nanoph-2023-0613_suppl_001.pdf]

Supplemental Information - Enhancement of the internal quantum efficiency in strongly coupled P3HT-C_{60} organic photovoltaic cells using Fabry-Perot cavities with varied cavity confinement

Lianne M.A. de Jong,[†] Anton Matthijs Berghuis,[†] Mohamed S. Abdelkhalik,[†] Tom P.A. van der Pol,[‡] Martijn M. Wienk,[‡] Rene A.J. Janssen,[*,‡] and Jaime Gómez Rivas[*,†]

†*Department of Applied Physics and Science Education, Eindhoven Hendrik Casimir Institute, and Institute for Complex Molecular Systems, Eindhoven University of Technology, P.O. Box 513, 5600 MB Eindhoven, The Netherlands*

‡*Department of Chemical Engineering and Chemistry, and Institute for Complex Molecular Systems, Eindhoven University of Technology, P.O. Box 513, 5600 MB Eindhoven, The Netherlands*

E-mail: r.a.j.janssen@tue.nl; j.gomez.rivas@tue.nl

S1. Optical constants of P3HT

In Fig. S1(a), the real and imaginary refractive index spectra of P3HT films can be found for different P3HT concentration. These spectra were obtained via ellipsometry measurements. In Fig. S1(b) the corresponding absorption coefficient of P3HT is given and can be compared to the absorption coefficient of C_{60}.

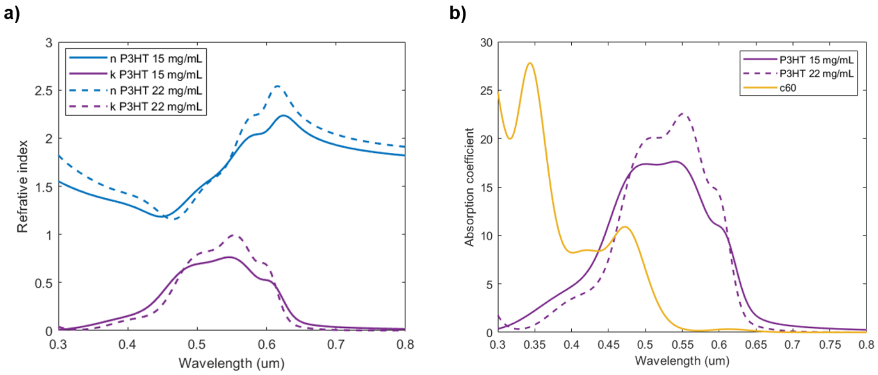

Figure S1: (a) Real and imaginary refractive index spectrum of P3HT. Measured via ellipsometry for a P3HT layer spin-coated using a solution with a concentration of 15 and 22 mg/ml. (b) Absorption coefficient of P3HT and C_{60}, calculated from the corresponding refractive index spectra.

S2. Additional dispersion measurements of the absorptance of the OPV cells

In Fig. S2, the measured and calculated absorptance of the reference cells, the Fabry-Perot cells with 10 nm mirrors and the Fabry-Perot cells with 30 nm mirrors can be found for different angles of incidence in a range between -40 and 40 degrees. For the calculated absorptance, the transfer matrix method (TMM) is used.

Part of the discrepancy between measurements and calculations is caused by the difference in substrate. In the case of the calculation, the glass substrate has an infinite thickness with the light source located inside the substrate, while for the measurement the light source is located in air, thus outside the substrate.

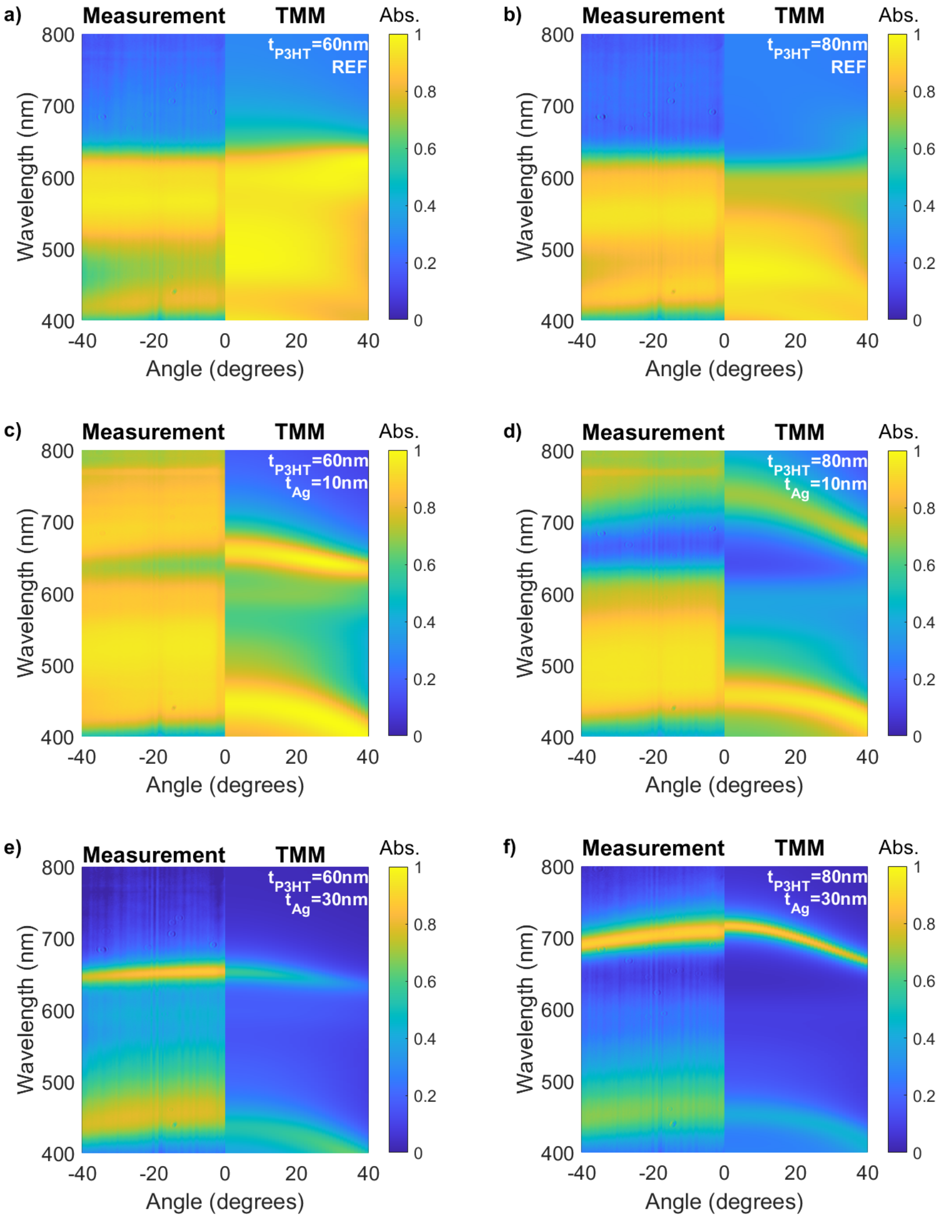

Figure S2: Measurements and TMM calculations of the angular absorption dispersion of P3HT-C_{60}-BCP solar cells. (a) reference cell $t_{P3HT} = 60$ nm. (b) reference cell $t_{P3HT} = 80$ nm. (c) Fabry-perot cell $t_{Ag} = 10$ nm $t_{P3HT} = 60$ nm. (d) Fabry-perot cell $t_{Ag} = 10$ nm $t_{P3HT} = 80$ nm. (e) Fabry-perot cell $t_{Ag} = 30$ nm $t_{P3HT} = 60$ nm. (f) Fabry-perot cell $t_{Ag} = 30$ nm $t_{P3HT} = 60$ nm. The FP cells show the formation of polariton bands, whose wavelengths decreases with increasing angle.

Additionally, small variations in the refractive index used for the calculations and the refractive index of the deposited material can exist. For the P3HT layer, the refractive index used in the calculations was determined via ellipsometry of spin-coated P3HT layers (See

section S1). This means the refractive index values are expected to be the same for measurement and calculation. However, the anisotropy of the P3HT layer and the difference between in-plane and out-of-plane refractive indices was not taken into account in the calculation.

For the thin silver film of the Fabry-Perot cells, the ellipsometry data of a uniform 100 nm thick silver film was used. The values obtained for this thicker film are only an estimation of the refractive index values of the silver films used in the measurement due to the non-uniform evaporated thin layers (see section S4). The difference is the largest for the 10 nm silver films, which explains the large discrepancy between calculation and measurement for these cells.

S3. JV-curves

Figure S3 shows all the JV-curve measurements of the four substrates, each containing four contacted cells.

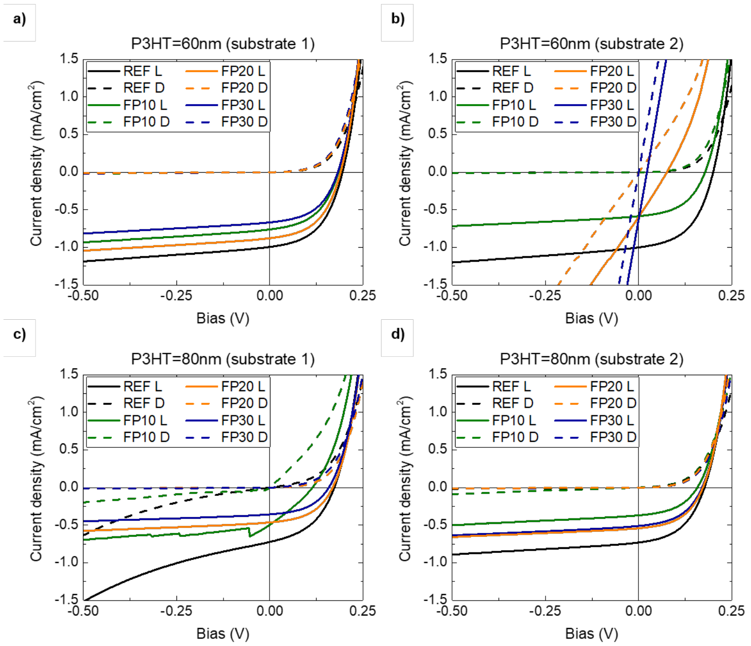

Figure S3: Additional JV measurements of the cells in the four different substrates.

S4. Scanning electron microscope images of thin film Ag (10 nm)

A scanning electron microscope (SEM) image is taken for the Ag film with 10 nm to check the deposited film quality. The SEM image (Fig. S4) shows a poor-quality film that forms islands instead of planar film. This film quality explains the poor results for the Fabry-Perot OPV cells with 10 nm Ag mirrors.

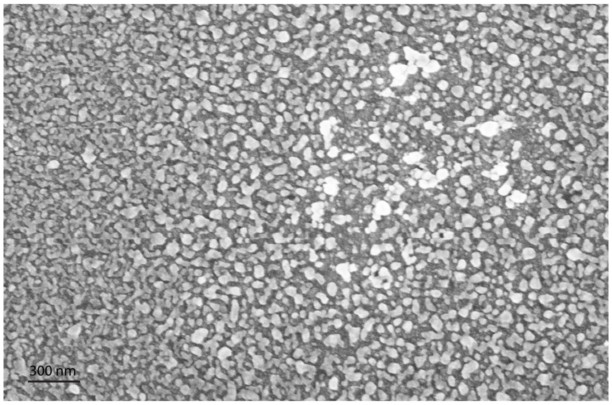

Figure S4: SEM image of a Ag film with a thickness of 10 nm evaporated onto an ITO/glass substrate.

S5. Electric field distributions

We compare the fields ins the different layers of the solar cell for the reference cell and the Fabry-Perot cavity cell using the transfer matrix method (TMM). For the FP cavity solar cell (20 nm Ag), we observe a large enhancement of the electric fields around the lower polariton wavelength and a slight enhancement of the fields at the UPB wavelength, while the field is reduced for other wavelengths compared to the reference.

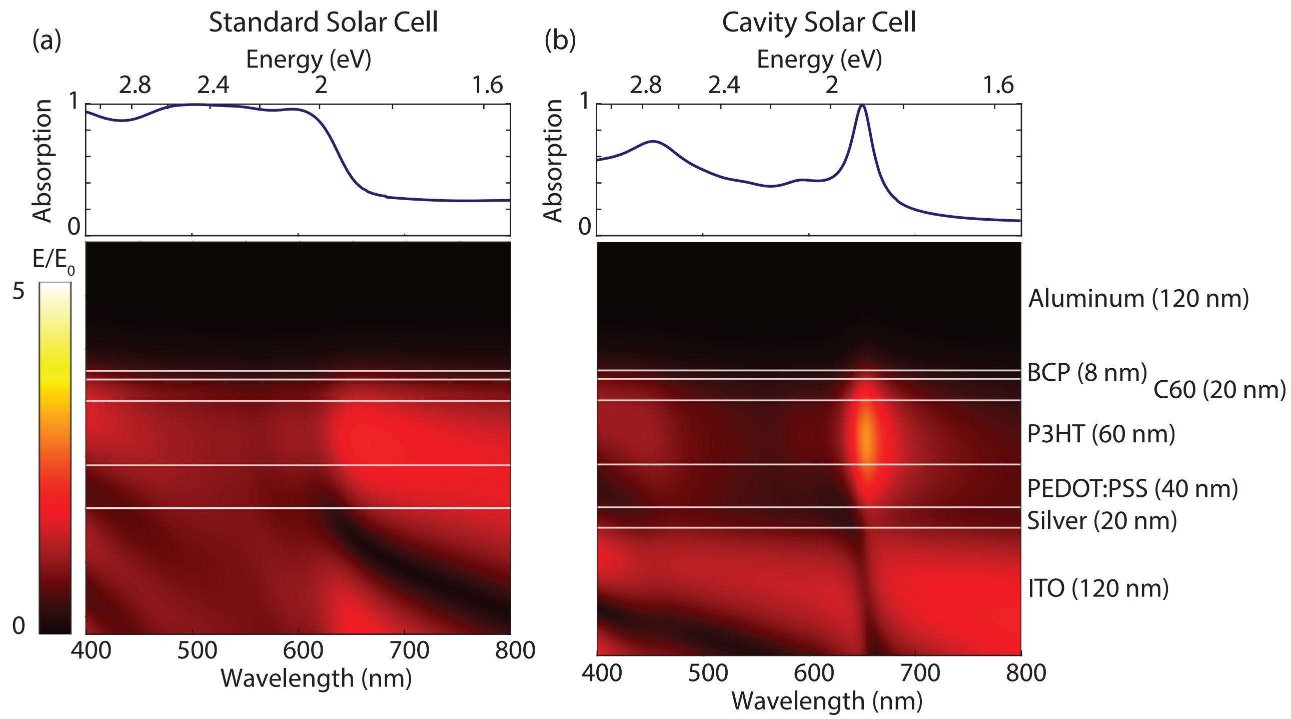

Figure S5: a. Wavelength-dependent field distribution and simulated absorption in a reference solar cell, and b. in a FP cavity solar cell with a 20 nm thick silver mirror.

S6. Low energy absorption tails

The low energy absorption tail of the EQE measurement of the P3HT-C_{60} solar cells with 60 nm P3HT thickness was shown in the main text. For reference, we include the low energy tail for cells with 70 nm P3HT thickness and 80 nm P3HT thickness in Fig. S6. In the case of a P3HT thickness of 80 nm, the lower polariton peak has an energy in the absorption tail, making an accurate analysis of the Urbach energy difficult. However, for the 70 nm P3HT layer cells similar results are found as for the 60 nm P3HT cells. The reduced Urbrach energies found for cells with 70 nm P3HT are presented in Fig. S7, together with the Urbach energies of the cells with 60 nm P3HT. The uncertainty in the Urbach energy is given by the uncertainty of the exponential decay fit.

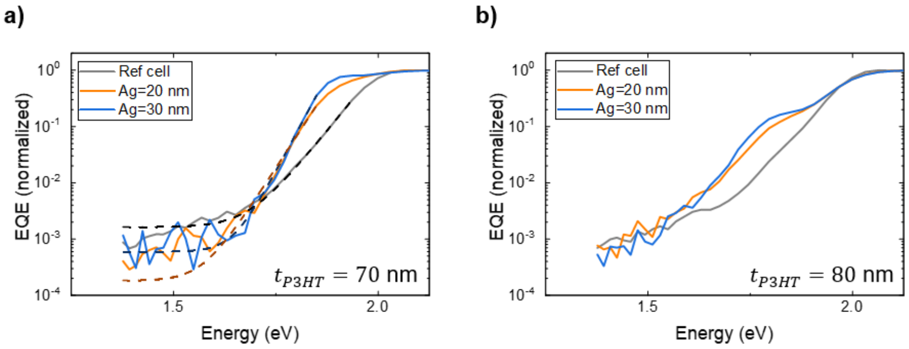

Figure S6: (a) Low energy tail of the measured EQE spectra of PV cells with a P3HT thickness of 70 nm, fitted using an exponential decay fit. The graphs show a steepening of the absorption edge in the case of strongly coupled Fabry-Perot cells. (b) Lower energy tail of the measured EQE spectra of PV cells with a P3HT thickness of 80 nm. The lower polariton peak is located in the absorption tail, making it difficult to determine the Urbach energy.

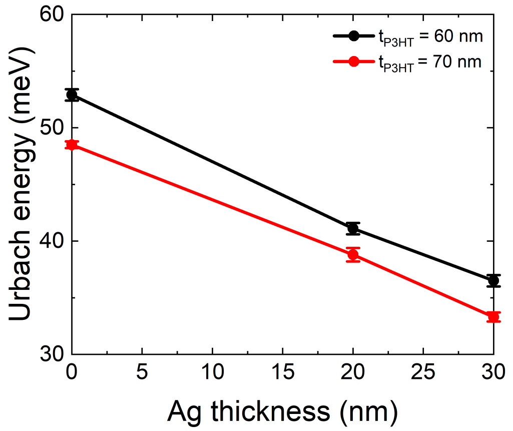

Figure S7: The Urbach energy, equal to the decay constant retrieved from the exponential decay fits (eq. (??)), plotted as a function of Ag film thickness for PV cells with a P3HT layer of 60 ad 70 nm.

S7. Bare Fabry-Perot resonances and uncoupled excitons

The energy of the Fabry-Perot resonance (E_{FP}) for different angles of incidence has been determined by fitting the bare cavity structure calculated using the TMM (see methods).

This fit was done in Matlab using the peak finding function. The result is shown in Fig. S8 for $t_{P3HT} = 80$ nm and $t_{Ag} = 20$ nm for s-polarization. In this figure, the fit is depicted by the green-dotted curve. The cavity loss, γ_{FP}, has been determined from the full width at half maximum of the FP resonance at normal incidence. For a P3HT layer of 80 nm and an Ag film thickness of 20 nm, it is equal to $\gamma_{FP} = 0.08$ eV. This value is used in the coupled oscillator model (see Methods), as it can not be directly retrieved from the measurements due to the P3HT exciton absorption.

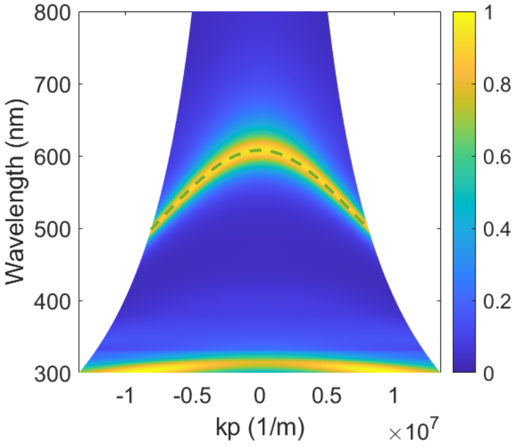

Figure S8: Fabry-Perot resonance fitted for a cavity thickness of 80 nm filled with a dielectric with the same real component of the refractive index as P3HT but without absorption, and an Ag film thickness of 20 nm. The fit is shown by the green dotted line.

To determine E_e and γ_e, the absorptance spectra of the reference solar cell without top Al contact are used. The top aluminum contact is removed from the structure because it causes a weak Fabry-Perot resonance. This resonance is relatively weak, but does significantly influence the peak energies. The exciton peak fits are shown in figure S9. The fit is done using a multiple peak fit function using Gaussians. The three main peaks, belonging to the excitons of P3HT, are indicated with orange, yellow and purple. The additional peaks are needed to correct for the absorption of the C_{60} and are not used in the coupled oscillator model. E_e are equal to the frequency of the peak's maxima, which differ slightly for different P3HT thicknesses. γ_e are determined from the full width at half maximum of the exciton peaks. For a P3HT layer of 80 nm, we obtain $E_{e1} = 2.6$ eV, $E_{e2} = 2.2$ eV, $E_{e3} = 2.0$ eV,

$\gamma_{e1} = 0.5$ eV, $\gamma_{e2} = 0.4$ eV, $\gamma_{e3} = 0.1$ eV.

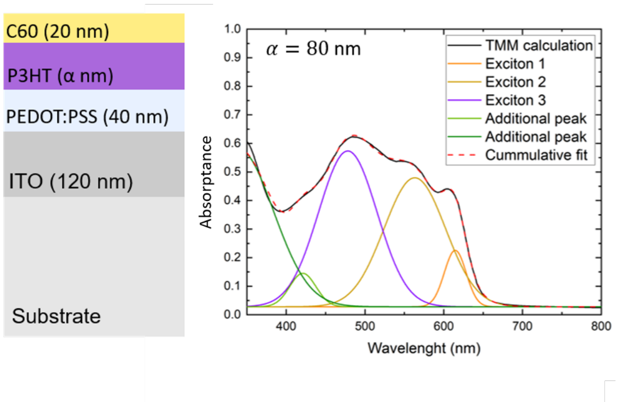

Figure S9: Fitted Exciton peaks of a full solar cell structure without top contact for a P3HT layer thickness of 80 nm.
